# Photon-pixel coupling: A method for parallel acquisition of electrical signals in scientific investigations

**DOI:** 10.1016/j.mex.2019.04.003

**Published:** 2019-04-24

**Authors:** Paul A. Gagniuc, Constantin Ionescu-Tirgoviste, Radu Gabriel Serban, Elvira Gagniuc

**Affiliations:** a Faculty of Engineering in Foreign Languages, Politehnica University of Bucharest, Romania; b National Institute of Diabetes, Nutrition and Metabolic Diseases “N.C. Paulescu”, Bucharest, Romania; c Center of Excellence in Translational Medicine, Fundeni, Romania; d University of Agronomic Sciences and Veterinary Medicine, Faculty of Veterinary Medicine, Bucharest, Romania

**Keywords:** Photon-pixel coupling, Parallel, Signal, Acquisition, Electrical activity, Darlington, Sensors

## Abstract

Here we describe a novel prototype method for parallel sampling of electrical signals from 200 sensors. The amplified signal from each sensor was remotely converted into a luminous signal on a LED matrix. A digital camera supported by a duralumin skeleton, was installed at 15 cm above an LED matrix inside an opaque box. Images were sampled at discrete time intervals of 5 s. A total of 25,920 images of the LED matrix have been recorded. Thus, 5.2 million measurements have been recorded as light intensities from the LED matrix. Light intensities of individual LEDs from the images were converted into 1 pixel value/LED. Each pixel value was then converted into percentages for evaluation. We used this methodology to measure the temporal variation of the electrical current on the skin of the torso on human volunteers, to assess the presence of a correlation between the electrical activity and diabetes (Ionescu-Tirgoviste et al., 2018). This method also allowed us to compile the first high resolution map of the electrical activity generated by the human skin (Ionescu-Tirgoviste et al., 2018).

• A novel method for a parallel acquisition of electrical signals which can be applied in any related field.
• It provides the ability to retrieve a large number of electrical channels simultaneously.
• It provides for an inexpensive and reliable way to digitize hundreds to thousands of channels at video rate frequencies.

## Graphical abstract

**Figure:**
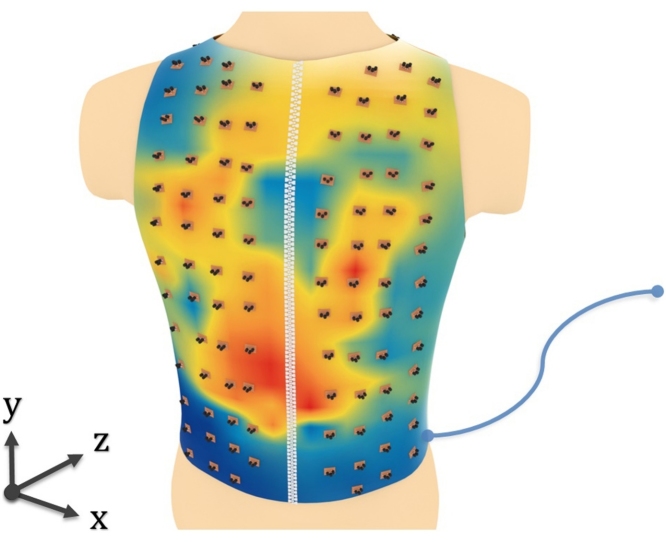

**Specifications Table**

**Table:** 

Subject Area:	• Engineering • Computer Science • Medicine and Dentistry
More specific subject area:	A parallel signal acquisition system for scientific investigations.
Method name:	Photon-pixel coupling
Name and reference of original method:	[1]
Resource availability:	All the data and solutions are described and included in this article.

## Method details

The skin is an organ of interest for novel investigative strategies. A large-scale prototype has been developed and used to retrieve 5.2 million measurements from the human skin. Based on these measurements, we developed the first high resolution map of the electrical activity generated by the torso [1].

**Note: Photon-pixel coupling** refers to a simultaneous chain conversion of electrical signals into pixel values. (ie. parallel conversion of electrical signals to light signals to pixel values to percentage values).

## Sensor internals

**Fig. 1:**
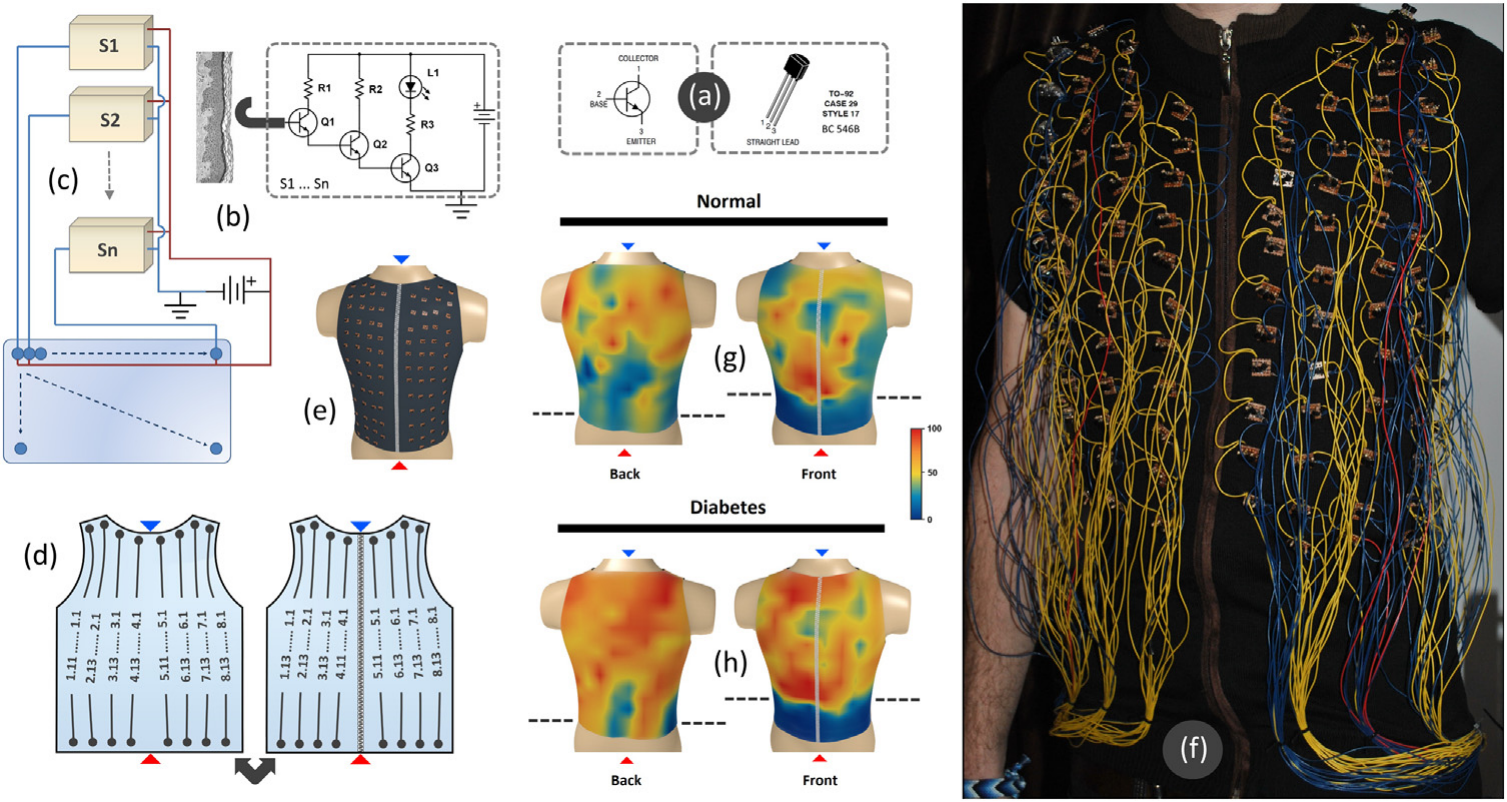
**Sensor internals and organization.** (**a**) The BC546B transistor pins and the case type. (**b**) The sensor diagram and a section through the epidermal layers. Electronic parts: Q1,Q2, Q3 (BC546B), R1(1 MΩ), R2 (100 kΩ), R3 (100 Ω) and L1 (Ø 5 mm, white light, Uf = 2.9 V). (**c**) A multi-sensor schematic, representing a vertical sensor line and the connection to the LED matrix (**d**) The organization of the vertical sensor lines on the ventral (right) and dorsal (left) surface of the vest. (**e**) physical location of sensors on the torso. The red triangle represents the bottom of the vest and the blue triangle represents the top of the vest. On each surface, the vertical sensor lines are noted from 1 to 8, starting from the left. The sensors on the vertical sensor lines are noted from 1 to 11 or 13 (starting from the bottom up). (**f**) The fully wired system of the vest prototype – front view. (**g**) distribution of measurements on the torax of normal individuals (**h**) distribution of measurements on the torax of diabetic individuals.

A total of eight electronic parts compose a sensor: three transistors (Q1,Q2, and Q3), an electrode wire and three resistors, namely R1(1 MΩ), R2 (100 kΩ), R3 (100 Ω) (Fig. 1a). We used discrete leaded components instead of microcomponents due to robustness and ease of handling. All of these components were attached to a printed circuit board (PCB) for rigidity (Fig. 1b). The eighth electronic component was an LED (measured parameters: Uf = 2.87V ± 0.06, C = 5 pF ± 2, Ir = 2 nA) that resided outside the PCB of the sensor. The internal structure of the sensors was a variation of a Darlington triplet [1]. Each sensor contained a number of three transistors in the emitter-base configuration for maximum amplification of the biological signals. The base current through the upper-left (Q1) transistor is amplified through the emitter. The emitter of the Q1 is then directly connected to the base of the Q2 transistor, where the current is again amplified.

The emitter of the Q2 is directly connected to the base of the Q3 transistor, where the current is amplified the third time. The BC546B is a general-purpose NPN bipolar junction transistor with a TO92 housing type. The transistor model BC546B was chosen for Q1–Q3 positions as an ideal candidate for stress endurance to electrical fluctuations (see the schematic diagram). In this specific context, the most relevant general characteristics of BC546B model are the Collector-Base Voltage (V_CBO_ = 80 V), Emitter-Base Voltage (V_EBO_ = 6 V), Collector-Emitter Voltage (V_CEO_ = 65 V), the DC Current Gain (h_FE_ = ˜180), Collector Current–Continuous (I_C_ = 100mAdc), P_total_ = 500 mW, Current–Gain–Bandwidth Product f_t_ = 300 MHz). Our measurements on the BC546B transistor characteristics indicate a hFE = ˜330 ± 40, Uf = 685 mV, Ube = 677 mV ± 0.08, and a Ic = 3 mA ± 0.5. Important note: The BC547A transistor (or equivalent models) was an alternative candidate. BC547A showed a very high sensitivity compared to the BC546B model. From our initial experiments, the prototype sensors based on the BC547A model allowed for contactless measurements, from a distance of 1–2 cm from the skin. However, the BC546B was chosen for a lower sensitivity, to avoid a signal contamination between sensors.

## Sensor organization

The vest is a type of sleeveless coat that hugs the human torso. A moderate wide mesh fabric (composed of 80% cotton, 15% Polyamide, and 5% Elastane) was chosen in order to allow the skin an optimal exchange with the environment (Fig. 1b). Sensors have been arranged on the outer part of the vest in vertical sensor lines (Fig. 1c). The vertical sensor lines (cords) were composed of sensors arranged linearly on the vest. The number of sensors that composed a vertical sensor line varied between 11 and 13 sensors in accordance with the anatomy of the human torso. Each sensor was rigidly placed on a PCB. Their individual PCBs were sewn to the vest to allow variation to stretching. Depending on the anatomy of the patient torso, the skin surface covered by the sensors was located between T1 and L4 or L5 vertebrae. The ventral side of the torso was covered by 8 vertical sensor lines, each with 11 to 13 sensors attached. The dorsal side of the torso was also covered by 8 vertical sensor lines, each with 11 to 13 sensors attached. Thus, the sensor labeling was made by using the number associated with the vertical sensor line, followed by the sensor number along the vertical sensor line (ie. 2.5 – denotes sensor 5 from the vertical sensor line number 2).

## LED array organization and signal encoding

**Fig. 2:**
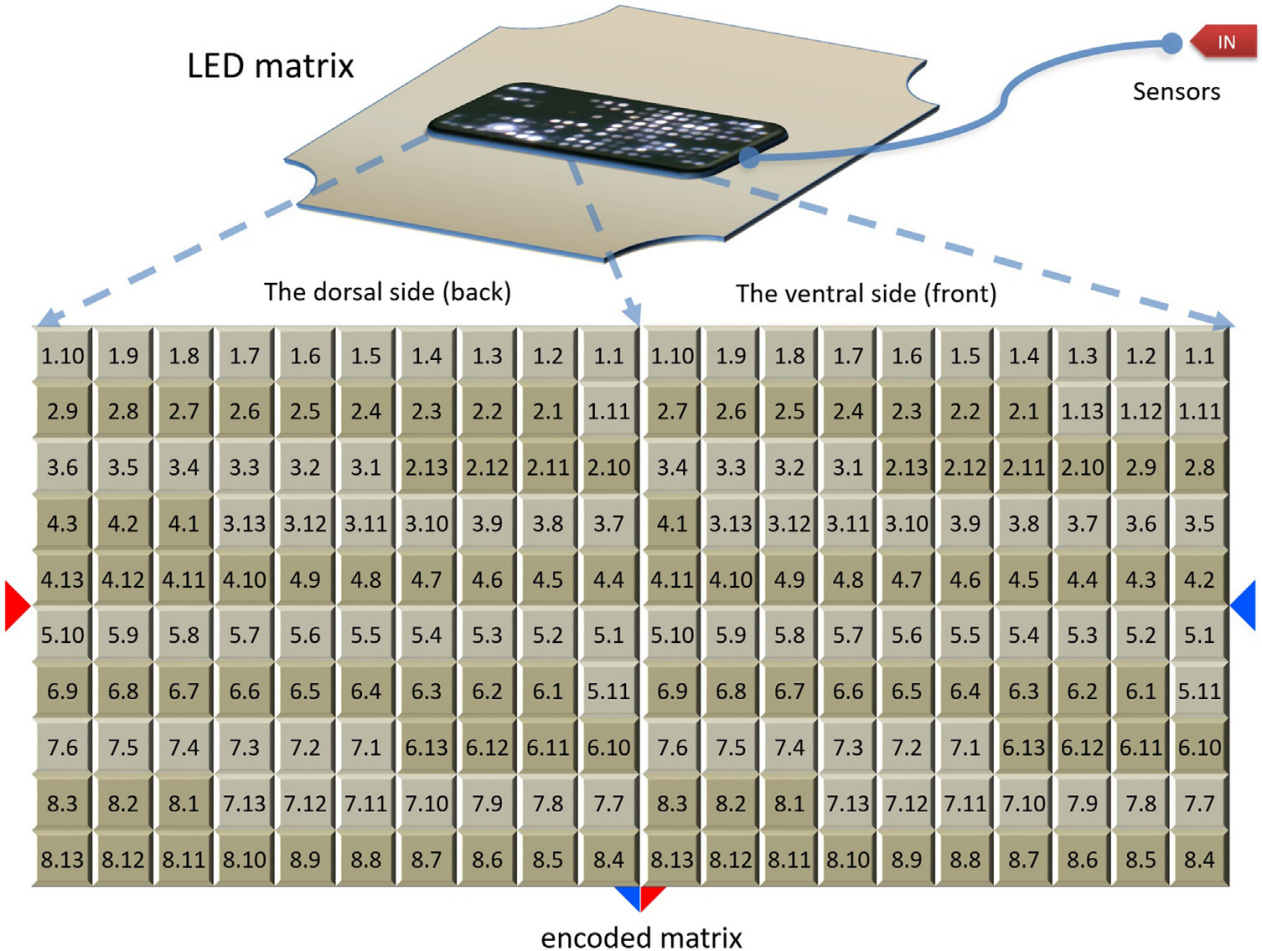
**LED array organization and signal encoding.** It shows the labeling and organization of the vertical sensor lines on the LED matrix. The right side of the LED matrix is associated with the dorsal side of the torso and the left side of the LED matrix is associated with the ventral side of the torso. The LED labeling was done by using the number associated with the vertical sensor line, followed by the sensor number along the vertical sensor line.

The LED array (or the LED matrix) was designed for a dimension of 20 × 10 elements which included a total of 200 LEDs (Fig. 2). The size of the LED matrix on the PCB was measured at 12 × 6 cm. In order to achieve a relative symmetry, the LED distribution was made by taking into consideration the ventral and dorsal face of the torso.

Thus, the left side of the matrix was associated with the dorsal area whereas the right side of the matrix was associated with the ventral area of the torso. Therefore, the first vertical half (left side) of this matrix incorporated 100 LEDs (10 × 10 elements) that had a correspondence to the vertical sensor lines from the dorsal side of the torso (Fig. 2). The second vertical half (right side) of the matrix also composed of 100 LEDs (10 × 10 elements), had a symmetrical correspondence to the vertical sensor lines from the ventral side of the torso (Fig. 2). Both on the right and on the left side of the LED matrix, the LEDs from the vertical sensor lines have been encoded (positioned) one after the other starting from the right to the left of the matrix. Note: As long as the correspondence between the sensors and the position of the LED is noted in advance, the order of the LEDs on the surface of the matrix may be chosen in an arbitrary manner.

## The electric diagram of the vest

The positive and negative terminals and a copper electrode were the main inputs for each of the 200 sensors (S_1,_ S_2_ … Sn, where n = 200). A Direct Current (DC) adjustable power supply (3 V up to 12 V) was connected to the first two inputs of the sensor (operational at 7.5 V). The sensors were connected in parallel on vertical sensor lines. Thus, each vertical sensor line was supplied with DC in parallel. The electrode of each sensor was a thick copper wire that penetrated through the vest material and made contact with stratum corneum (the surface of the skin). Thus, each electrode sampled the local electrical current into the base of the Q1 transistor for further amplification. The output of each sensor was the collector of the Q3 transistor which was further connected to a Light Emitting Diode (LED). The LED was positioned outside the sensor on a 200 Light Emitting Diode array. The connection between the sensor and the LED was made with an insulated copper wire. The lenght of each copper wire that made the connection between a LED and a sensor has varied between 50 cm and 1 m, depending on the vertical position of the sensor on the vertical sensor line. A total of 32 copper wires fed 16 sensor lines (16 vertical sensor lines × 2 copper wires = 32 wires). Of these 32 wires, 16 wires have been connected to the negative terminal (−) and the other 16 wires to the positive terminal (+) of the DC power supply. The negative terminal (−) of the DC power supply was connected directly to the Earth (direct physical connection to the Earth). In this case, the Earth served as a constant potential reference, against which the biological potentials were measured. Furthermore, the positive terminal (+) of the DC power supply was connected to the vertical sensor lines as well as to the LED matrix. The cathode of all LEDs from each line of the matrix was connected with a wire to the positive terminal (+) of the DC power supply. The anode of each LED was individually connected to the collector of a Q3 transistor of a corresponding sensor. The negative terminal (−) was grounded and connected only to the vertical sensor lines (to the emitter of each Q3 transistor). A total of 200 insulated copper wires made the electrical connection between the PCB of the sensors and the appropriate LEDs (200 sensors × 1 copper wire = 200 copper wires). Thus, a total of 232 copper wires (32 + 200 = 232 copper wires) made the electrical connection between the vest and the outside environment, namely the black box.

## The black box

**Fig. 3:**
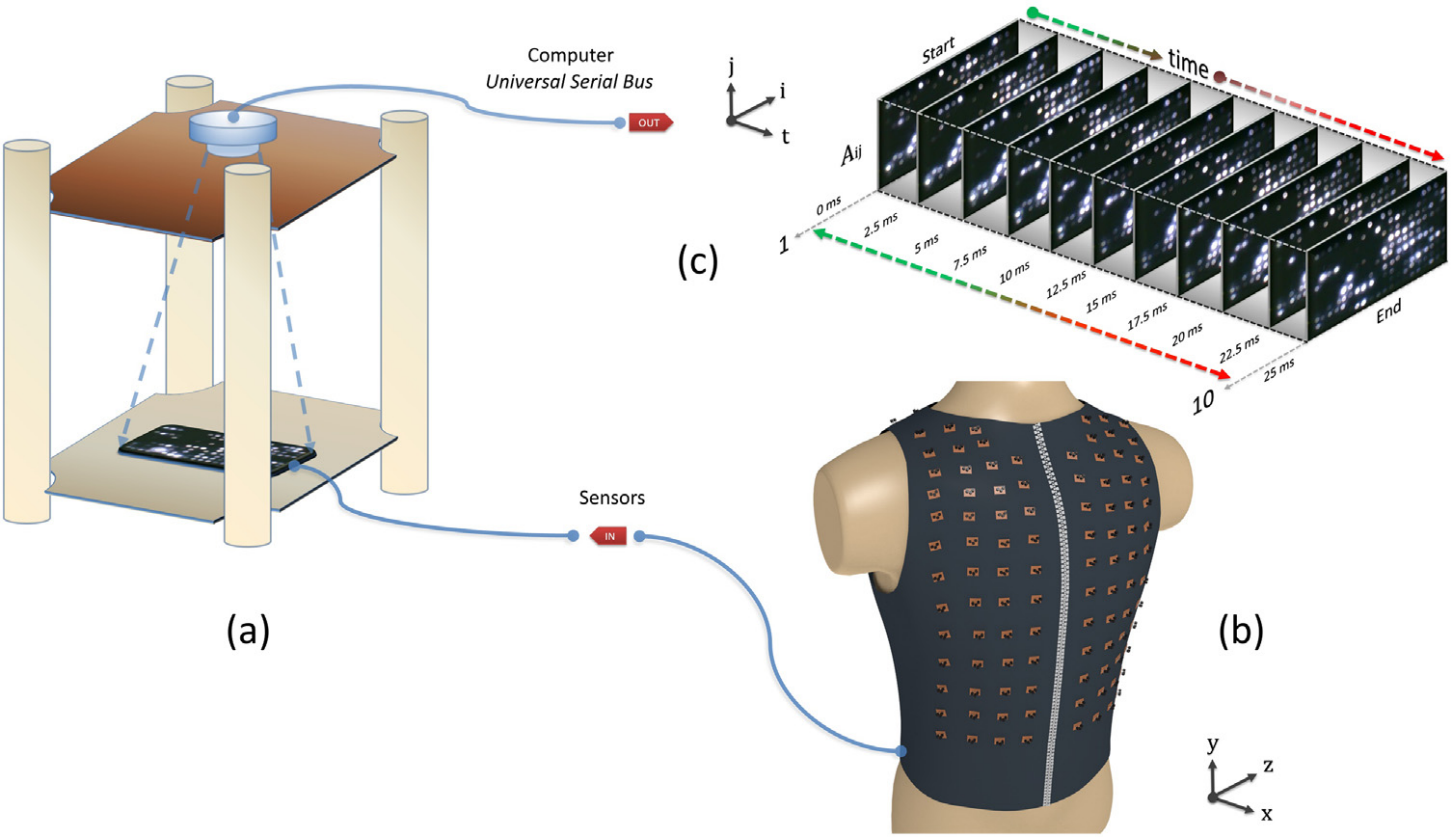
**The black box.** (**a**) A video camera targeted on the LED matrix in a dark environment of a sealed box. (**b**) The ventral side of the electrodermal vest. The image shows 100 sensors arranged in 8 vertical sensor lines attached to the vest. (**c**) Examples of image samples taken over time inside the black box. On the three axes, i represents the columns of the LED matrix, j represents the LED matrix lines and t represents the measuring time interval.

The main strategy in the experiment consisted in making parallel measurements of the electrical signals that arrived from the sensors (Supplementary material 1). Fundamentally, a video camera is a fast signal multiplexer. The light intensity of individual LEDs was detected by a digital camera which in turn directly indicated the amplified biological signal. Therefore, the black box was made of a cardboard box (23 × 23 × 50 cm) whose interior was isolated from the ambient light. The interior contained a duralumin skeleton, which supported a digital camera focused on the LED array (Fig. 3a). A filter made from a semi translucent plastic sheet (thickness: 0.5 mm) was placed above the LED matrix to avoid the contamination of light signals from neighboring LEDs. Three main cables connected the black box with the external environment, namely an output cable and two input cables. The output cable of the video camera communicated with the computer via the Universal Serial Bus (USB). The first input cable was a thick cord made of 232 tightly wrapped copper wires. Of which, 200 wires were responsible for bringing the amplified electrical signals from each sensor to the LEDs, and 32 wires were responsible for the power supply of the vertical sensor lines (Fig. 3a, b). The cable of the DC power supply represented the second input of the black box. Some parameters of the box have been adjusted before the measurements were made, such as the distance on the z-axis between the digital camera and the LED array, the position on the x-axis and the y-axis of the digital camera above the LED matrix, the focus of the digital camera or the optimum voltage (7.5 V) applied to the vertical sensor lines.

The size of the LED matrix on the PCB was measured at 12 × 6 cm. Thus, for maximum clarity and correct image framing, the LED matrix was centered and the distance between the video camera and the LED matrix was set at 15 cm. After these adjustments, the position of the video camera has been fixed with silicone for a permanent position, and the black box was sealed. Thus, the black box has allowed for an isolated and constant environment during all experiments. The list of materials used in the prototype design and construction is detailed in Table 2.

## Experiment design

**Fig. 4:**
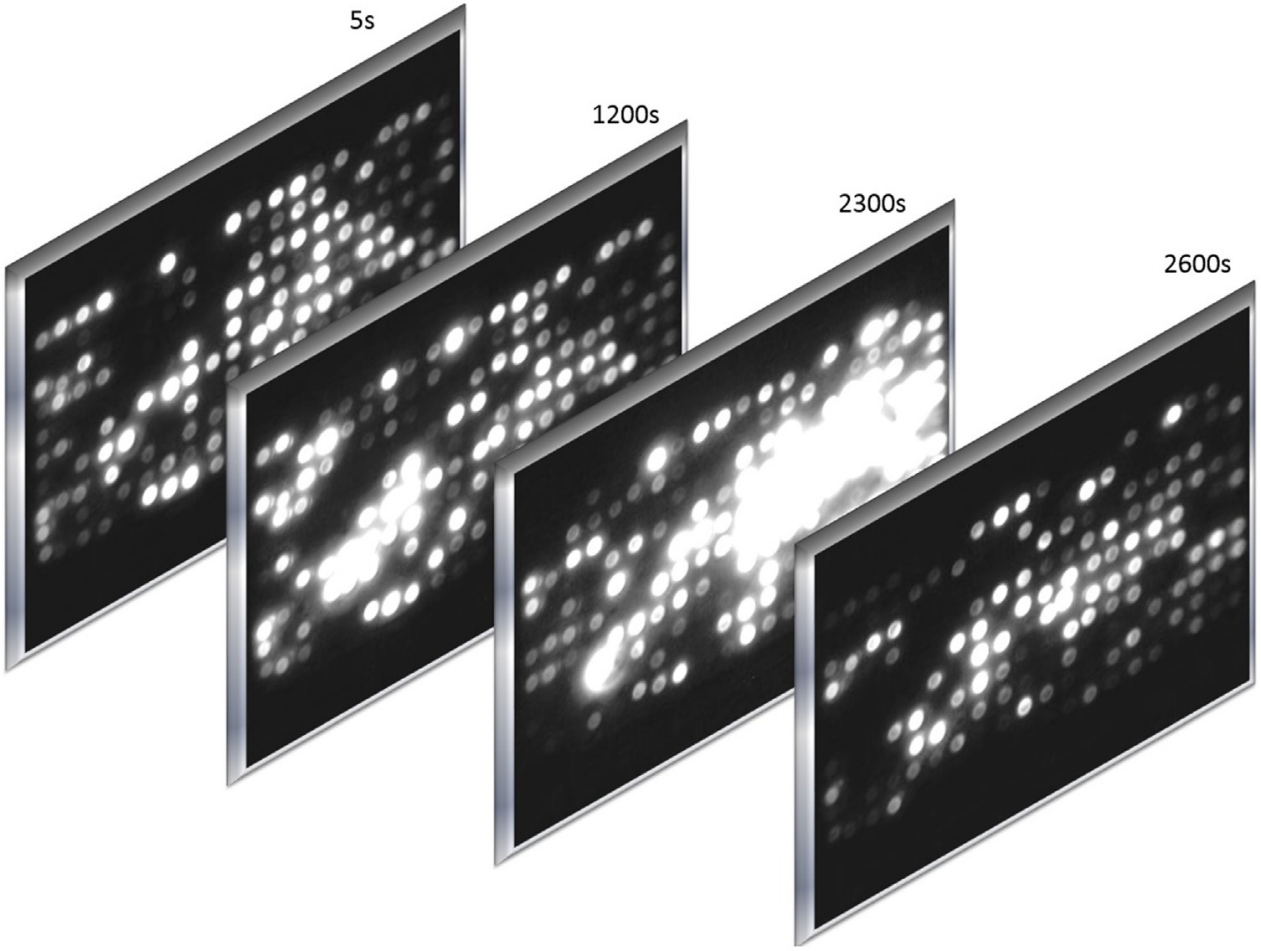
**Examples from a diabetic subject.** Electrical activity patterns on the LED matrix at different time intervals.

A total of 36 volunteers were asked to participate in the experiment in a dry fasting state [1]. All experiments were performed in accordance with relevant guidelines and regulations. The methods were carried out in accordance with the approved guidelines. Prior to the experiment, all subjects signed an informed consent. All experimental protocols were approved by the Ethical Committee of “NC Paulescu” Institute of Diabetes. Before and during the experiments an air conditioner and a dehumidifier were used to maintain constant temperature (25 °C) and relative humidity (40%). The relative humidity was maintained at low levels in order to avoid an increase in the apparent temperature of the body, thus hindering the evaporation of perspiration from the skin. At an air temperature of 25 °C and a relative humidity of 40%, the absolute humidity is 9.2 g/m^3^ and the dew point temperature is 10 °C. During the experiments, the subjects had to wear the vest and sit comfortably and immobile on a chair without a backrest. To avoid an alteration of the electrical potential, the body of the subjects were isolated from the ground whereas the negative terminal (−) of the sensors was grounded. Each experiment was conducted in the morning, within a period of one hour (3 subjects/day). During this interval, two oral doses of 37 g glucose have been administered to the subjects in order to observe the metabolic response. The first dose was administered after 15 min and the second dose after 30 min (75 g in total). Before each experiment, the electrodes of the sensors were individually disinfected with clotrimazole and alcohol, and tested for correct functionality. As the experiments unfolded the patients were carefully monitored by a physician and a dose of injectable insulin was always available. However, such an intervention was not necessary. A short video representing the LED matrix of a diabetic subject, for a period of 1 h, can be found in Supplementary material 2. Another example of a dynamic signal pattern over time can be seen in Fig. 4.

## Data acquisition

A dedicated opensource software designed by us for the video camera of the black box, was responsible for retrieval and storage of images (Supplementary material 3). These images represented the state of the LED matrix at discrete time intervals. Our dedicated application for the video camera allows for wide measurement intervals (3 s up to 60 s) and the experiment duration can be set from 3 min to 120 min. The optimal setting for our experiment has been set to 1 h (60 min) with an interval of 5 s, which resulted in a constant series of 720 images/subiect:

$$Tot\left(i\right)=\frac{number\;\text{ of}\;\text{ seconds}\;\;\text{ in}\;\text{ one}\;\;hour}{measurement\;\text{ interval }}=\frac{\left(60\;\min\times 60\;\sec\right)}{5s}=720\;\;images$$

Where Tot represents the total number of images and i represents an index for each subject. Since the intensity of the LEDs can be deduced at any resolution, a small resolution of 320 × 240 pixels was used in order to obtain a more accurate capture of the light signals. These images were stored on the computer in the Joint Photographic Group (JPG) format at 24b for further analysis. For screenshots and additional explanations on the software applications used by the “Vesta” project, please see Supplementary material 4.

**Note:** The maximum number of signal samples that can be taken in parallel with this method is directly proportional to the frame rate of the detector, namely the video camera. The number of images taken by the video camera per second is known as: frames per second (FPS). Frame rates generally range from 15 fps to 120 fps. This suggests that a high number of electrical signals can be sampled in parallel, with a sampling rate of 15 Hz–120 Hz. Thus, the number of measurements would be equal to Ns = n × f, where Ns represents the total number of measurements, n the total number of sensors monitored in parallel and f, the fps of the video camera, or another discreet preset. For instance, our discreet preset for f was 1 frame at 5 s. Thus, in our experiment Ns = 5.2 million measurements. For experiments requiring a high sampling frequency, high-speed cameras can be used. High-speed cameras are capable of capturing moving images with frame rates higher than 250 fps, which in turn indicates a 250 Hz parallel sampling or higher [2].

## Calibration and output conversion

Two types of calibrations were performed. The first calibration was made to adjust the physical positions of the LEDs from the PCB to ideal coordinates. The second calibration was made in order to equalize the amplification gain of the sensors.

### Calibration of LED positions

**Fig. 5:**
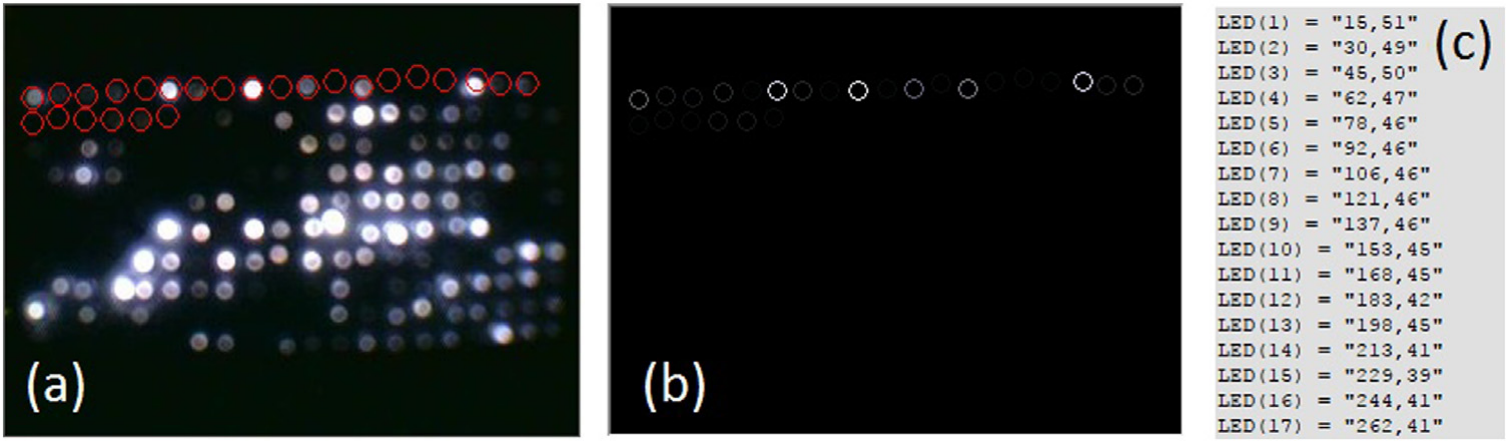
**The vector map.** (**a**) The pixel coordinates of each LED manually selected by us. The selection was made on the brightest pixel of each LED, from left to right on each row. Here, for exemplification purposes the panel shows just 25 selected positions (red circles). (**b**) The intensity of the pixel associated with each LED is indicated by the brightness of the perimeter of each circle. (**c**) The vector map in the form v ^(n)^ = [x, y], where x and y represent the coordinates of each pixel associated with a LED and n represents the number of the LED in the LED matrix.

An early stage of the analysis has taken into account the detection of the LEDs coordinates from the images collected by the black box (Fig. 5a–c).

A separate software module was specifically designed for this operation and the coordinates of the LEDs were stored in vector format in order to be further used by the main analysis software (Supplementary material 5 and 6). In practice, the LEDs may not be perfectly aligned on the PCB array, however, the coordinates stored in vector map format allowed for a re-calibration of the LEDs positions. In this manner, the intensity of a LED was represented by one pixel value taken from the center of the LED from the image (Fig. 5a–c).

### Conversion of light to numerical values

For the output of the sensors we used white LEDs which allowed the examination of a gradient of pixels, between pure black and pure white (Table 1). The brightness of the LEDs from the captured images has been translated directly into numerical values by converting the pixel values from the center of the LEDs to percentage values, were pure black = 0%, pure white = 100% and intermediate values for the gradient between the two. (Supplementary material 6).

**Table 1:** The list of electronic components which comprise the sensors. The table shows the associated values for the labels shown on the schematic diagram of the sensor and the total number of electronic parts used in this prototype.

Components	Notation	Parameters	Number of parts per sensor	Total number
Transistor	Q1 … Q3	BC546B	3	600
Resistor	R1	1 MΩ	1	200
Resistor	R2	100 kΩ	1	200
Resistor	R3	100 Ω	1	200
Light-emitting diode	L1	Ø 5 mm, white light, Uf = 2.9 V	1	200
Antenna electrode	–	L = 2 cm, U-shaped, Ø 0.5 mm	1	200
Total electronic components			8	1600

**Table 2:** Macro components. The list of materials used in the prototype design and construction.

Components	Parameters	Number of parts per sensor	Total number
Copper wires	L = ˜360 m	5	1
Power supply	3 V–12 V, 350 mA – Typically used 7.5 V	–	1
Digital video camera	320 × 240 × 24b	–	1
Over-elastic vest	80% cotton, 15% Polyamide, 5% Elastin	–	1
Dark box	23 × 23 × 50 cm	1	1
PCB (LED)	25 × 25 cm	1	2
PCB (Sensors)	2 × 2 cm	1	200
Total Macro components			207

## Calibration of the sensor amplification

**Fig. 6:**
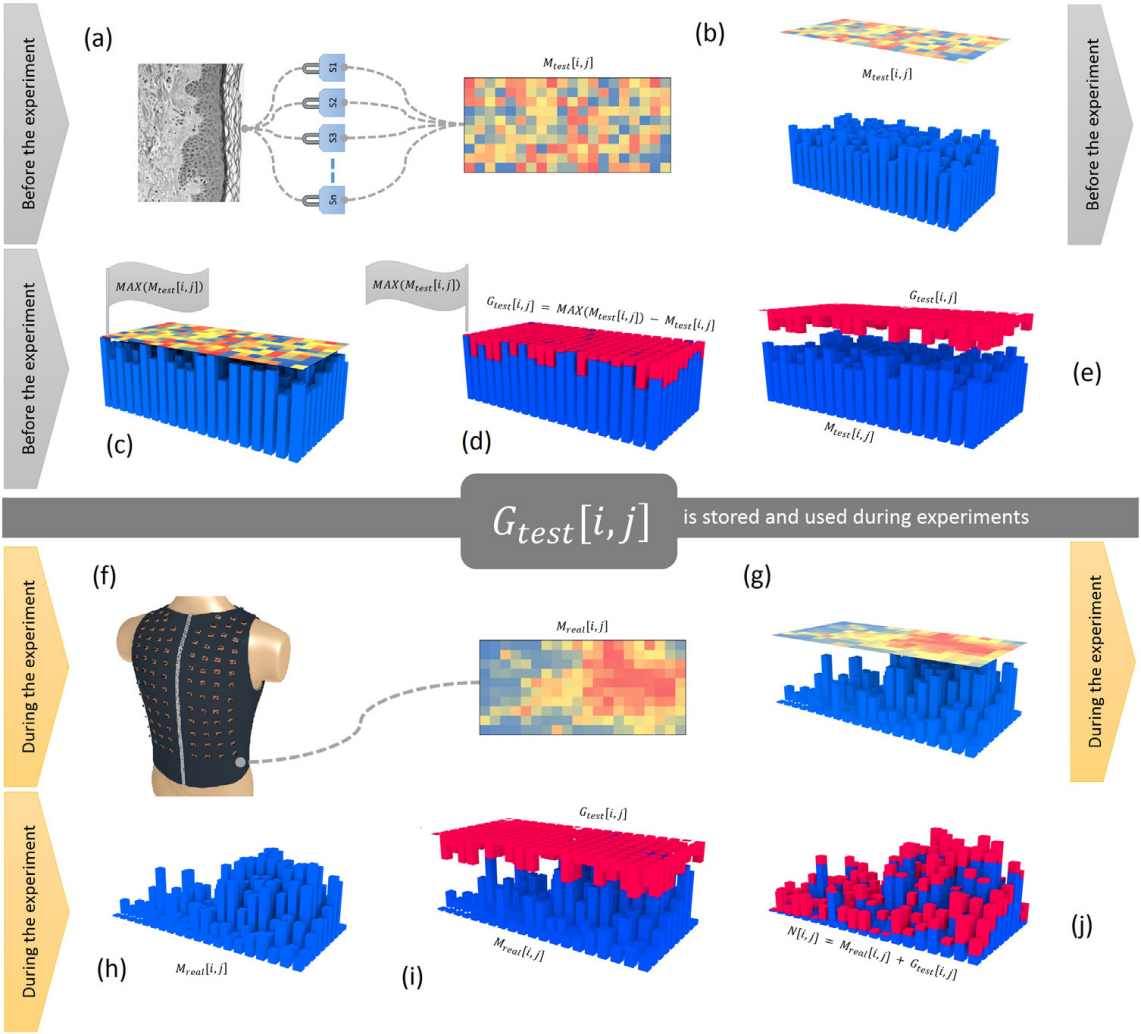
Calibration of the sensor amplification. (**a**) It shows the 200 sensors (Sn = 200) connected to a common signal – a point on the skin, and the M_test_[i, j] matrix which stores the numerical values of the parallel measurement. Inside the heatmap matrix, dark red represents a value of 100% and dark blue represents 0%. (**b**) The M_test_[i, j] matrix is represented in 3D space by means of blue bars whose height reflects the value of each cell from the heatmap matrix. (**c**) Shows the highest bar (MAX(M_test_[i, j])) on the 3d representation of the heatmap matrix. (**d**) Graphically shows the calculation of the gain matrix (G_test_[i, j]), whose bars are represented in red. The height of the red bars is represented by the space taken from any blue bar (M_test_[i, j]) up to the highest blue bar (MAX(M_test_[i, j])). (**e**) It shows a separation of the G_test_[i, j] matrix from the mold matrix, namely M_test_[i, j]. The G_test_[i, j] matrix is stored for later use. (**f**) It shows the 200 sensors positioned on different points on the skin of the torso, indicating that each sensor measures its own signal, and the M_real_[i, j] matrix which stores the numerical values of the parallel measurement. (**g**) The M_real_[i, j] matrix is represented in 3D space by means of blue bars whose height reflects the value of each cell from the heatmap matrix. (**h**) The 3d representation of the M_real_[i, j] heatmap matrix. (**i**) It shows an perfect overlap between the M_real_[i, j] matrix and the G_test_[i, j] matrix. (**j**) Graphically displays the calibration process and the N[i, j] matrix formation by adding the G_test_[i, j] values to the M_real_[i, j] matrix.

Prior to their attachment to the vest, the sensors were labeled and positioned close to each other. The electrodes of all sensors were connected together by short wires to a central electrode (a 1 mm thick wire); that received the signal from a single point on the skin (in our case the tip of a finger). This approach allowed for a simultaneous amplification of a single signal (Fig. 6a).

The numeric values from the output of each sensor were stored electronically in a matrix of 20 × 10 elements (M_test_[i,j], i = 1, …, 20, j = 1, …, 10). The output of each sensor was slightly different due to normal manufacturing variations in the electronic parts. A second matrix (G_test_[i,j]) of the same size as the first, was built to store the offset values from the maximum value found on M_test_[i,j] (Fig. 6b–e). The elements of this second matrix (G_test_[i,j]) were constructed from the maximum value found in the first matrix (MAX(M_test_[i,j])) minus the homologous value in the first matrix (M_test_[i,*j*]).

$$G_{test}[i,j]\;=\;MAX(M_{test}[i,j])\;-\;M_{test}[i,j]$$

**Note:** For the calibration process, the signal strength was less important since we were only interested in a common signal to indicate the amplification differences between sensors. The rationale behind the calibration method suggested that the gain difference between sensors was constant regardless of the common signal strength.

During the experimental measurements, the sensors were positioned equidistantly on the surface of the vest and the amplified signals were stored in the M_real_[i,j] matrix (Fig. 6f). In order to perform the calibration, the values from the G_test_[i,j] matrix remained constant throughout the experiment and were added to the values from the corresponding elements of M_real_[i,j] each time a new parallel measurement was made (Fig. 6g–j).

$$N[i,j]\;=\;M_{real}[i,j]\;+\;G_{test}[i,j]$$

Where N[i,j] contains the calibrated sensor values, M_real_[i,j] contains the real numerical values provided by the sensors during the experimental measurements and G_test_[i,j] stores the gain for each sensor (Fig. 6j).

**Note:** The initial labeling of the LEDs from 1 to 200 also allows a calibration by using a row vector (i = 1, …, 200), for instance:

Prior to the experimental measurements:

$$G_{test}\left(i\right)=Max(M_{test})-M_{test}(i)$$

During the experimental measurements:

$$N\left(i\right)=M_{real}\left(i\right)+G_{test}\left(i\right)$$

Where N[i] contains the calibrated sensor values, M_real_[i] contains the real numerical values provided by the sensors during the experimental measurements and G_test_[i] stores the gain for each sensor.

In this manner, an equal gain was obtained between sensors. Nevertheless, our main target was represented by the shapes created by the bright LEDs at the surface of the matrix. Thus, in our case the calibration did not make an important difference in the outcome of the experiment as long as the parameters were constant (ie. no power supply variations, no sensors replacement or no changes in the position of the sensors during experiments). However, in critical areas of science the above calibration is of great importance for the accuracy of the measurements. For instance, a correlation between a variety of sensor types, implies a normalized gain.

## Prediction for diabetes onset

**Fig. 7:**
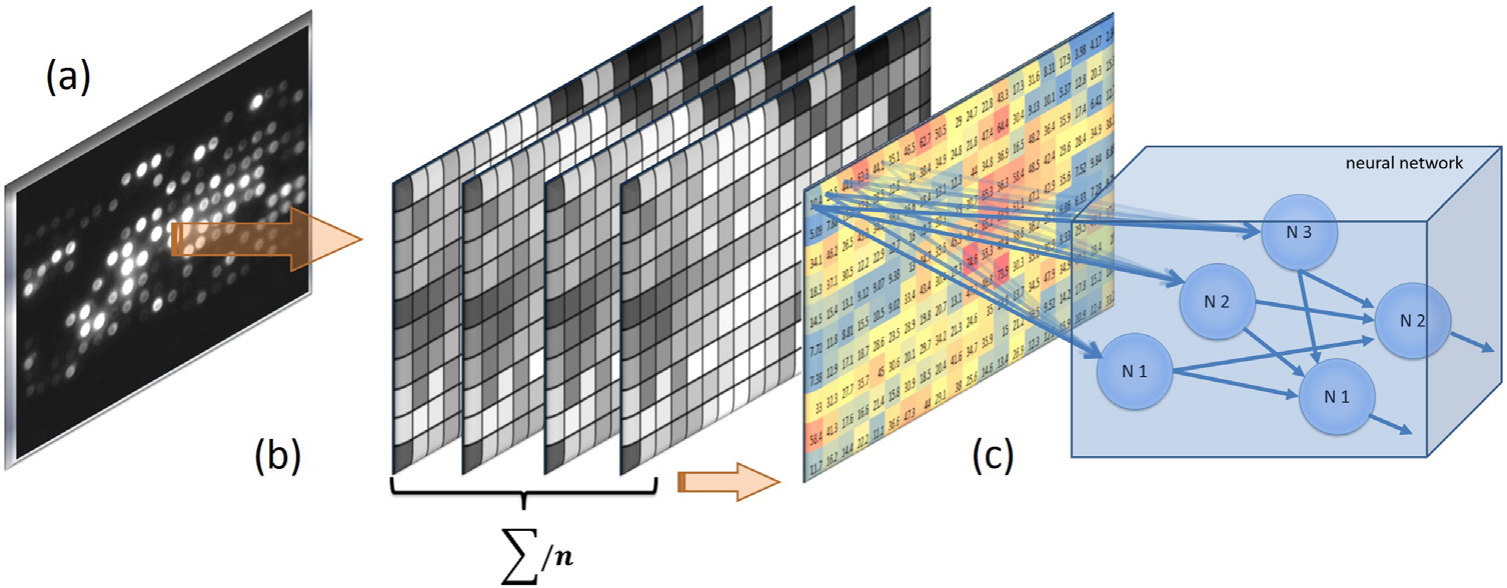
**From electrical signals to data.** (**a**) Acquisition of light signals from the LED matrix in the form of images. (**b**) Conversion of LED intensity to pixel values and percentages. The re-calibration of the LEDs positions was made using a vector map. (**c**) Data analysis and TensorFlow prediction based on the average matrix (the heat map) calculated between the elements of multiple matrices of a subject.

An application was developed with the goal of predicting the advancement of diabetes in an individual by analyzing their corresponding electrical activity heatmap (Supplementary material 7). For this software prototype we have used deep learning neural networks for classification of new patients in one of the two categories, namely normal or diabetes. Initially, the neural network has been trained using 36 images, each representing the average encoded matrix of an individual (Fig. 7a–c). These were divided into 5 healthy female subjects, 8 diabetic female subjects, 13 healthy male subjects and 10 diabetic male subjects. Each model was trained in 3 ways: once only on female subjects, once only on male subjects and finally on all the data. Following the training process, four new prediabetic patients were measured in order to test the prediction capabilities of the network. Measurements obtained from the new patients were used as the unknowns for the neural network input. For each patient, the values along the elements of 20 matrices (4000 measurements) were reduced to a single average encoded matrix (the heatmap). The average encoded matrix represented the equivalent of 100 s of measurements for a single patient. Based on our observations, measurements over 100 s did not lead to radical predictive improvements. Next, the average encoded matrix was given to the neural network as an image consisting of 10 rows and 20 columns of colored rectangles. The selected color matrix was then transformed into a matrix of depth 1 which contained values between 0 and 1.

This transformation was done by reversing the 3-color-scale conversion used to create the picture. The intermediate values indicated by the neural network can be considered as the trust value of the network, under 50% meaning healthy and over 50% meaning different degrees of predisposition for type 2 diabetes. The application and the details of this method are broadly presented in Supplementary material 4 and 7. Data obtained with this method is widely described in [3].
