# Supplementary material for: Photon-pixel coupling: A method for parallel acquisition of electrical signals in scientific investigations
Source: MethodsX. 2019 Apr 24;6:968–79. doi: 10.1016/j.mex.2019.04.003 (PMC6503210; doi:10.1016/j.mex.2019.04.003)
Supplement: Supplementary file 1 [file mmc1.pptx]

## Slide 1
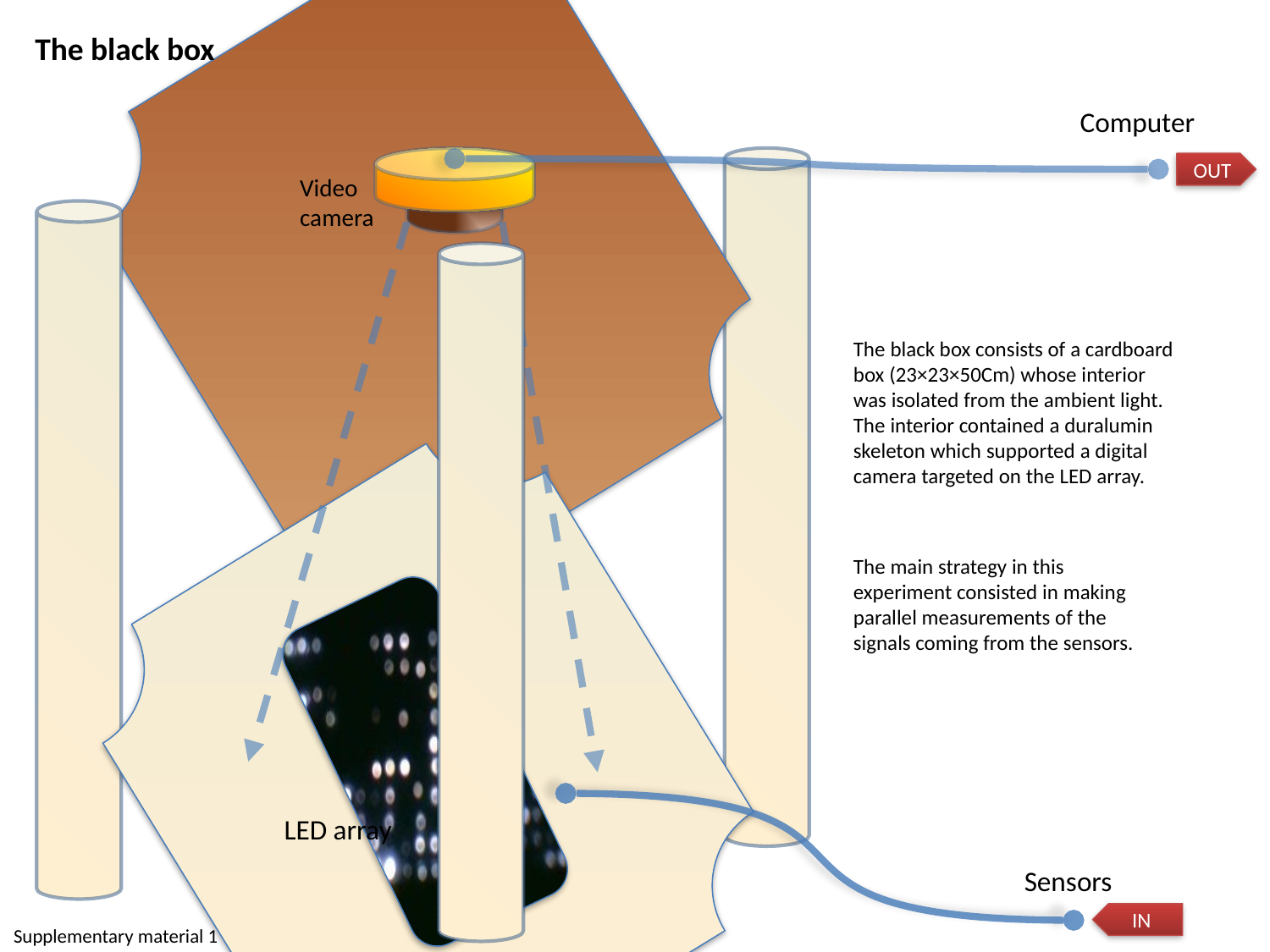

The black box
Computer
OUT
Video
camera
The black box consists of a cardboard box (23×23×50Cm) whose interior was isolated from the ambient light. The interior contained a duralumin skeleton which supported a digital camera targeted on the LED array.
The main strategy in this experiment consisted in making parallel measurements of the signals coming from the sensors.
LED array
Sensors
IN
Supplementary material 1

## Slide 2
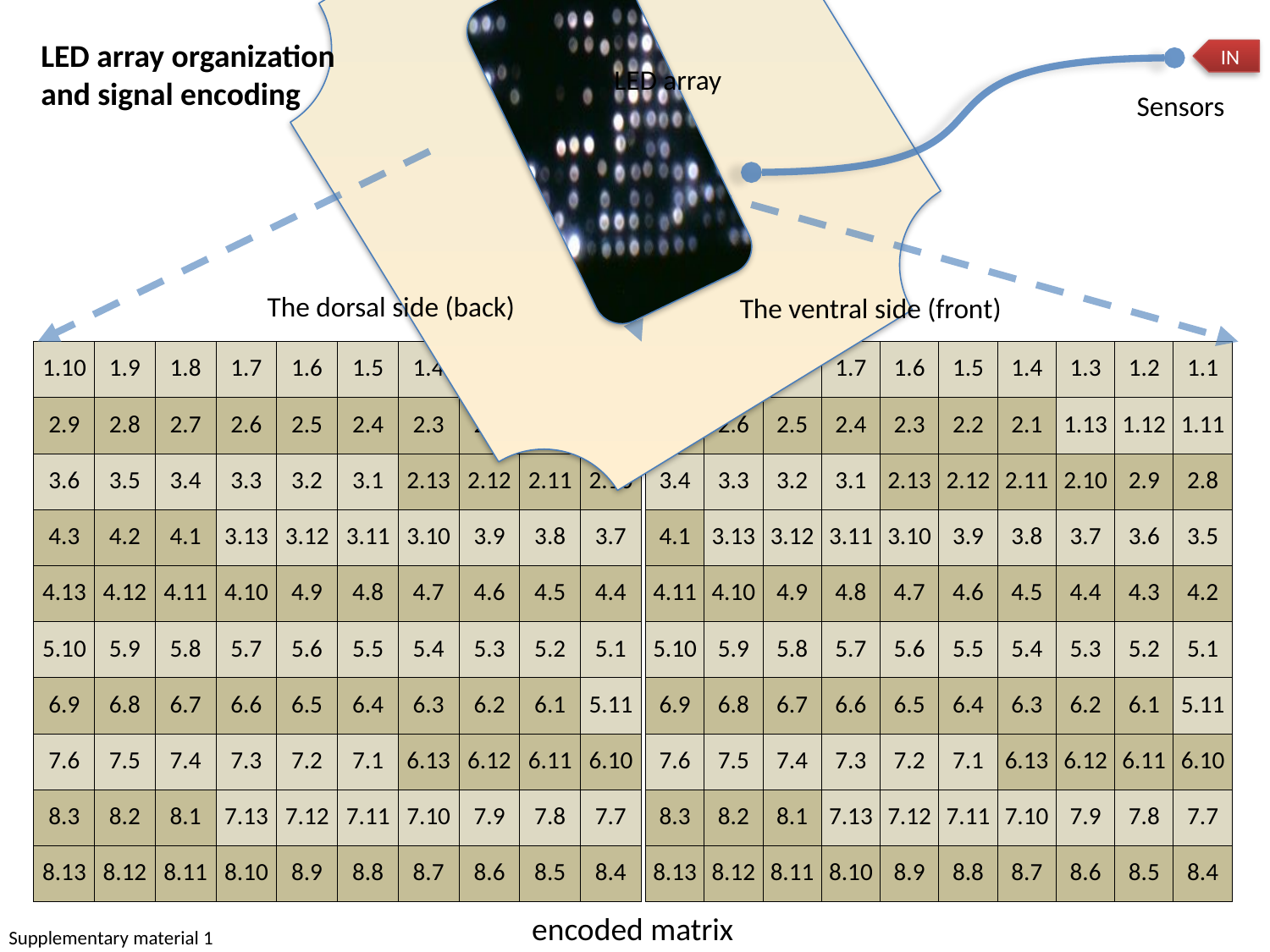

LED array organization
and signal encoding
IN
LED array
Sensors
The dorsal side (back)
The ventral side (front)
| 1.10 | 1.9 | 1.8 | 1.7 | 1.6 | 1.5 | 1.4 | 1.3 | 1.2 | 1.1 |
| --- | --- | --- | --- | --- | --- | --- | --- | --- | --- |
| 2.9 | 2.8 | 2.7 | 2.6 | 2.5 | 2.4 | 2.3 | 2.2 | 2.1 | 1.11 |
| 3.6 | 3.5 | 3.4 | 3.3 | 3.2 | 3.1 | 2.13 | 2.12 | 2.11 | 2.10 |
| 4.3 | 4.2 | 4.1 | 3.13 | 3.12 | 3.11 | 3.10 | 3.9 | 3.8 | 3.7 |
| 4.13 | 4.12 | 4.11 | 4.10 | 4.9 | 4.8 | 4.7 | 4.6 | 4.5 | 4.4 |
| 5.10 | 5.9 | 5.8 | 5.7 | 5.6 | 5.5 | 5.4 | 5.3 | 5.2 | 5.1 |
| 6.9 | 6.8 | 6.7 | 6.6 | 6.5 | 6.4 | 6.3 | 6.2 | 6.1 | 5.11 |
| 7.6 | 7.5 | 7.4 | 7.3 | 7.2 | 7.1 | 6.13 | 6.12 | 6.11 | 6.10 |
| 8.3 | 8.2 | 8.1 | 7.13 | 7.12 | 7.11 | 7.10 | 7.9 | 7.8 | 7.7 |
| 8.13 | 8.12 | 8.11 | 8.10 | 8.9 | 8.8 | 8.7 | 8.6 | 8.5 | 8.4 |
| 1.10 | 1.9 | 1.8 | 1.7 | 1.6 | 1.5 | 1.4 | 1.3 | 1.2 | 1.1 |
| --- | --- | --- | --- | --- | --- | --- | --- | --- | --- |
| 2.7 | 2.6 | 2.5 | 2.4 | 2.3 | 2.2 | 2.1 | 1.13 | 1.12 | 1.11 |
| 3.4 | 3.3 | 3.2 | 3.1 | 2.13 | 2.12 | 2.11 | 2.10 | 2.9 | 2.8 |
| 4.1 | 3.13 | 3.12 | 3.11 | 3.10 | 3.9 | 3.8 | 3.7 | 3.6 | 3.5 |
| 4.11 | 4.10 | 4.9 | 4.8 | 4.7 | 4.6 | 4.5 | 4.4 | 4.3 | 4.2 |
| 5.10 | 5.9 | 5.8 | 5.7 | 5.6 | 5.5 | 5.4 | 5.3 | 5.2 | 5.1 |
| 6.9 | 6.8 | 6.7 | 6.6 | 6.5 | 6.4 | 6.3 | 6.2 | 6.1 | 5.11 |
| 7.6 | 7.5 | 7.4 | 7.3 | 7.2 | 7.1 | 6.13 | 6.12 | 6.11 | 6.10 |
| 8.3 | 8.2 | 8.1 | 7.13 | 7.12 | 7.11 | 7.10 | 7.9 | 7.8 | 7.7 |
| 8.13 | 8.12 | 8.11 | 8.10 | 8.9 | 8.8 | 8.7 | 8.6 | 8.5 | 8.4 |
encoded matrix
Supplementary material 1

## Slide 3
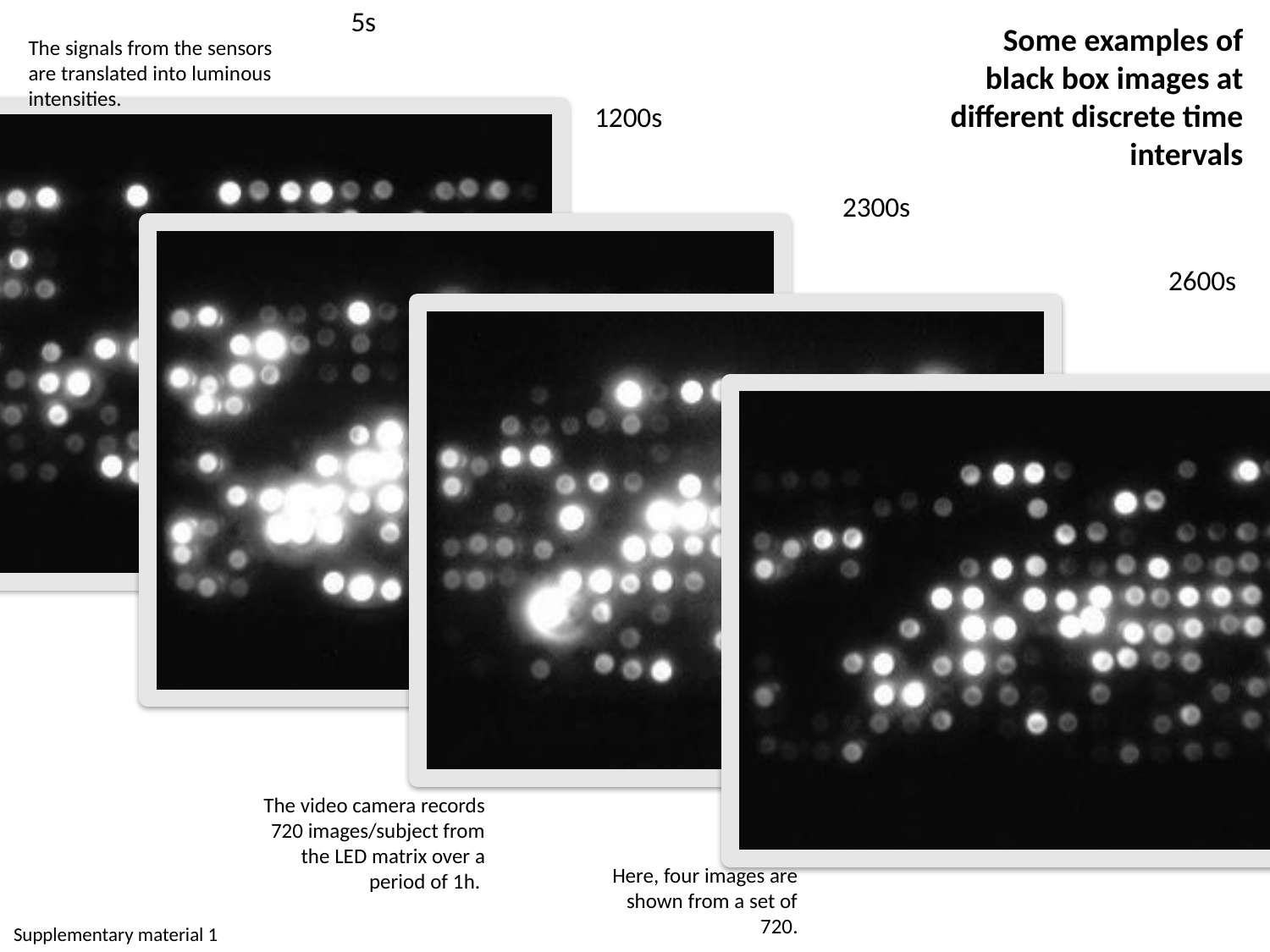

5s
Some examples of
black box images at
different discrete time
intervals
The signals from the sensors are translated into luminous intensities.
1200s
2300s
2600s
The video camera records 720 images/subject from the LED matrix over a period of 1h.
Here, four images are shown from a set of 720.
Supplementary material 1

## Slide 4
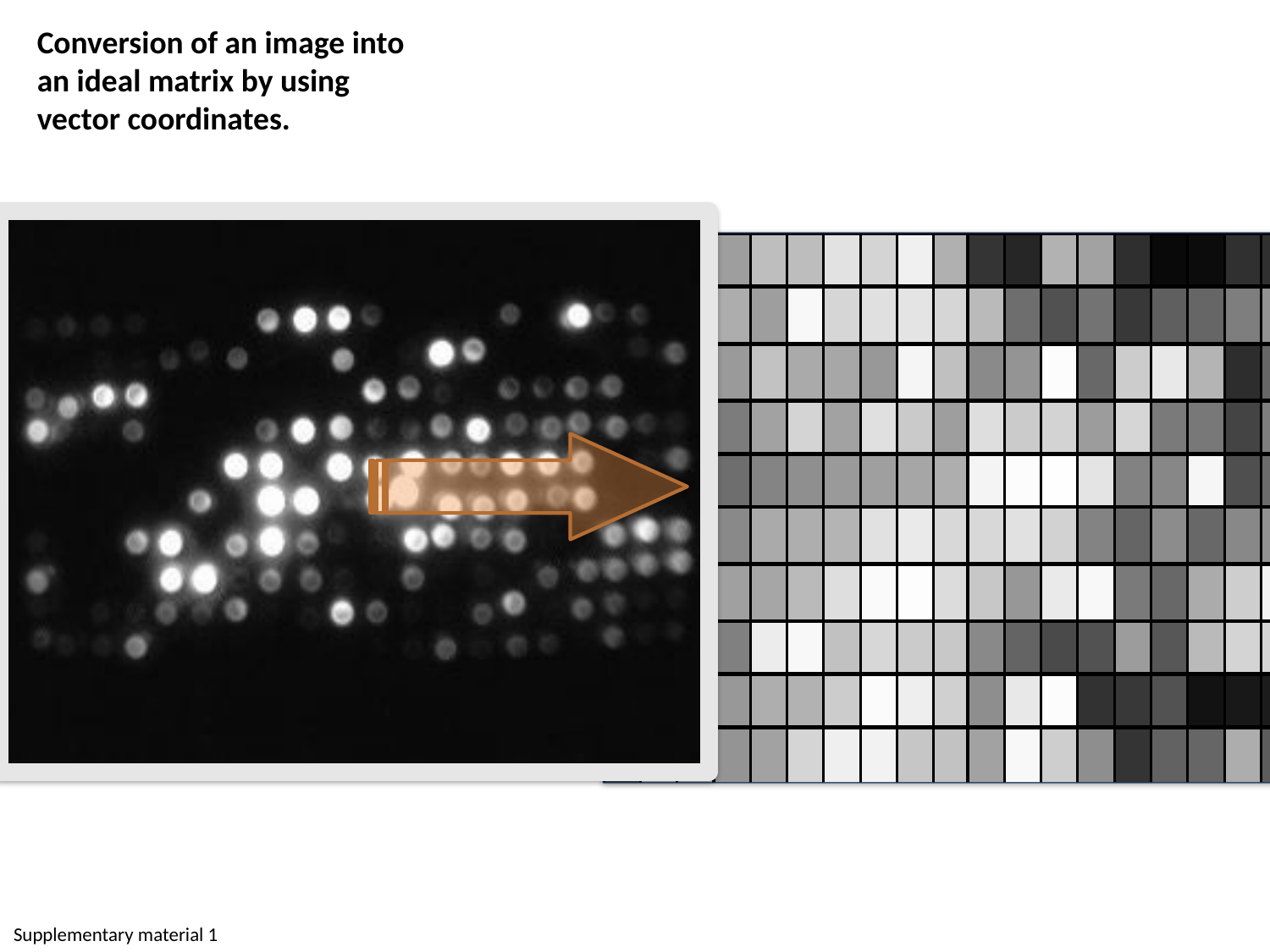

Conversion of an image into
an ideal matrix by using
vector coordinates.
Supplementary material 1

## Slide 5
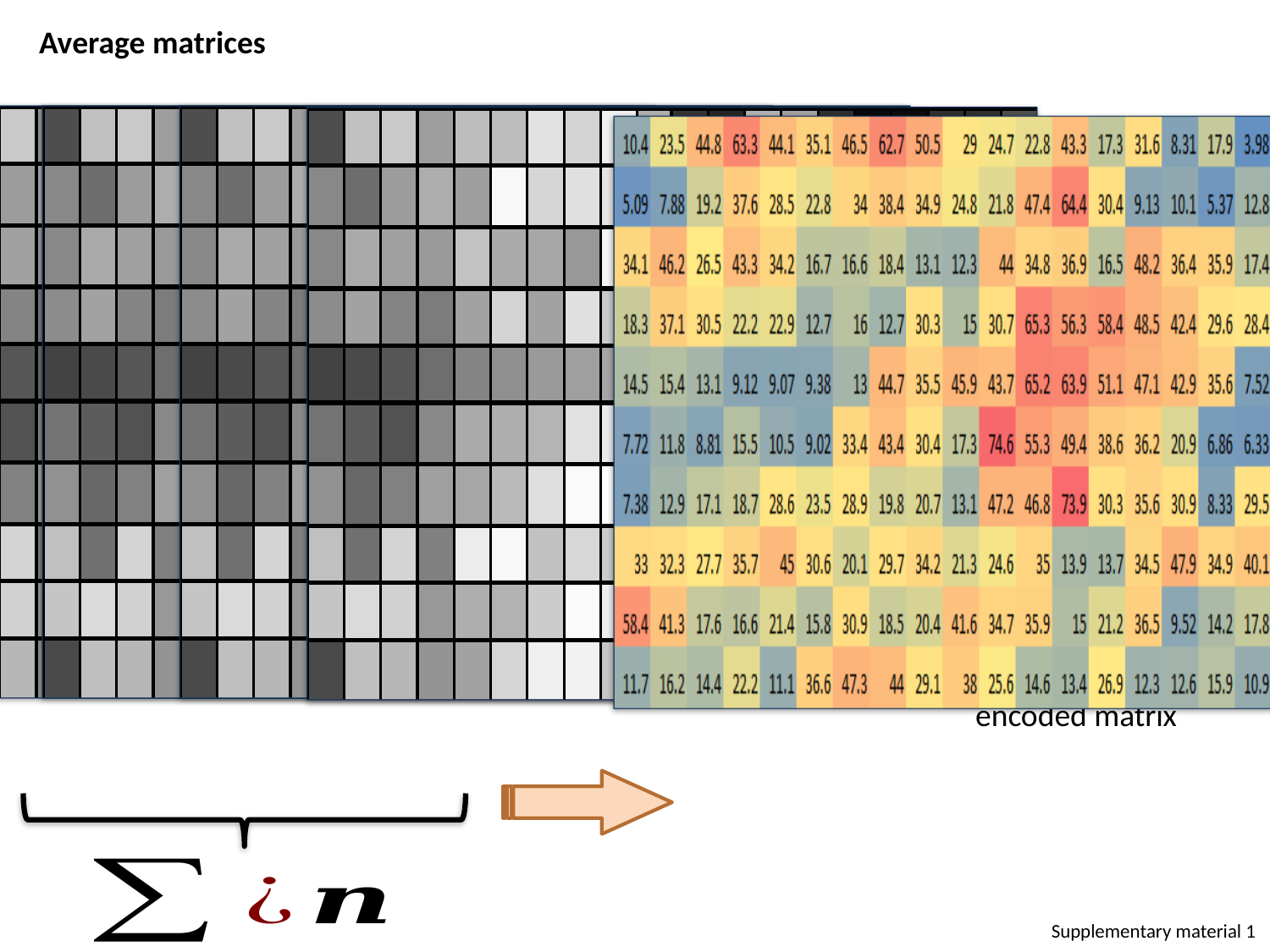

Average matrices
Average
encoded matrix
Supplementary material 1

## Slide 6
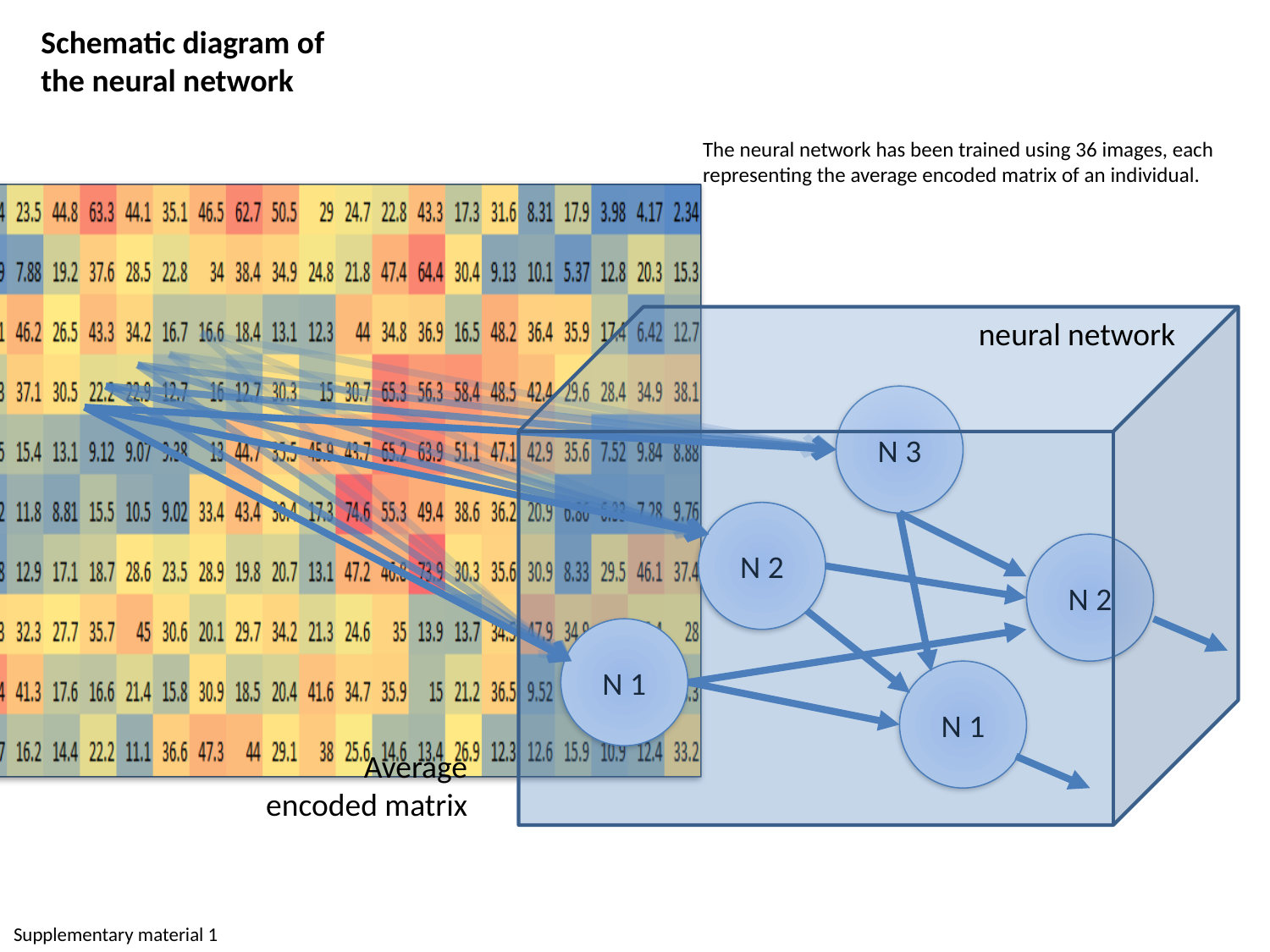

Schematic diagram of
the neural network
The neural network has been trained using 36 images, each representing the average encoded matrix of an individual.
neural network
N 3
N 2
N 2
N 1
N 1
Average
encoded matrix
Supplementary material 1
