# Supplementary material for: Photon-pixel coupling: A method for parallel acquisition of electrical signals in scientific investigations
Source: MethodsX. 2019 Apr 24;6:968–79. doi: 10.1016/j.mex.2019.04.003 (PMC6503210; doi:10.1016/j.mex.2019.04.003)
Supplement: Supplementary file 3 [file mmc3.docx]

Data acquisition

Our dedicated application for the video camera allows wide measurement intervals (3s up to 60s) and the experiment duration can be set from 3 min up to 120 min (Additional materials 4). The optimal setting for our experiment has been set for 1h (60 min) with an interval of 5 seconds, which resulted in a constant series of 720 images/experiment:

$$Tot=\frac{(60\min\times60 sec)}{5s}=720 images$$

Where *Tot* represents the total number of images / subject. Since the intensity of the LEDs can be deduced at any resolution, a small resolution of 320x240 pixels has been used in order to obtain a more accurate capture of the signals. These images have been stored in the application folder under “measurements” subfolder, in JPG (Joint Photographic Group) format at 24b for further analysis.


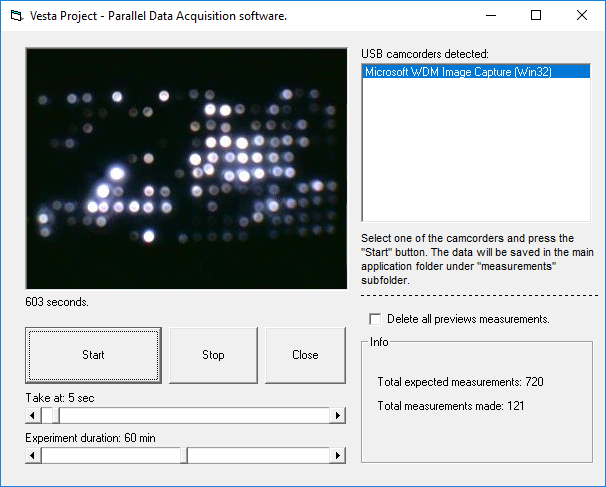


Post processing

LED coordinates

An early stage of the analysis has taken into account the detection of the LEDs coordinates from the images collected by the black box (Additional materials 5). Our strategy has allowed a preservation of the specific brightness of the LEDs and the elimination of barrel, pincushion and mustache optical distortions. A separate software module has been used specifically for this operation and the coordinates have been stored in vector format in order to be further used by the main analysis software (see Data retrieval). In practice, the LEDs may not be perfectly aligned on the PCB array, however, the vector map allows for a re-calibration of the LEDs positions. In this manner, the intensity of an LED was represented by one pixel value from the center of the LED.


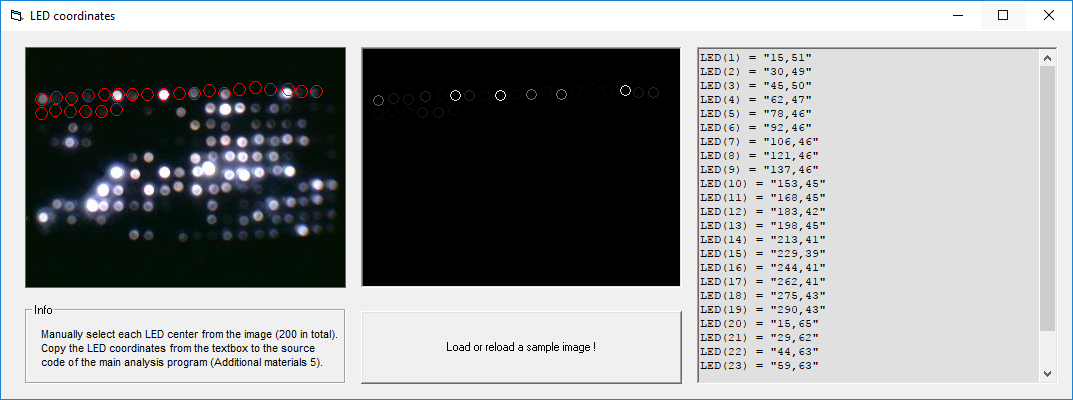


Data retrieval

The maximum brightness of individual LEDs has been collected from the images based on vector coordinates (Additional materials 6). The brightness of individual LEDs was converted to numeric values (between 0% - black and 100% - white). Thus, each image had a matrix correspondent. Next, each new matrix was appended as text (compatible with copy / paste in Excel) in the file: "folder name" dot "txt" found in the application folder.

The main application concerned several approaches:

1. Collecting the values from the images and the averages between matrices:

1. LED brightness (the conversion of each image into a matrix).
2. Mean LED brightness / subject (the mean over the elements of the matrices of each subject).
3. Mean LED brightness / group (the mean over the elements of the matrices in a group).

2. To quantify changes over time, the matrix was analyzed based on global brightness:

1. Average brightness over time (an average of the matrix elements in each step).
2. Up vs. down - average brightness over time (the duration of the increase or decrease of the light intensity).


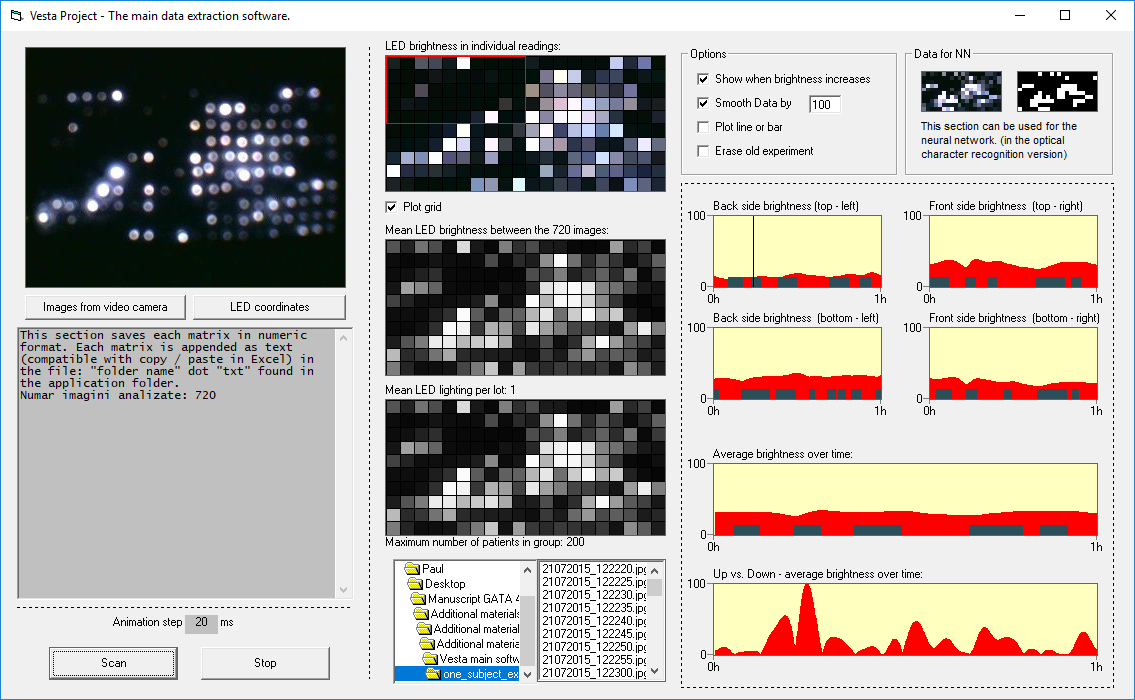


3. For a subtle determination of change, the matrix was also analyzed on four distinct regions:

1. Back side brightness (top - left).
2. Back side brightness (bottom - left).
3. Front side brightness (top - right).
4. Front side brightness (bottom - right).

4) Options for data smoothing have been implemented in order to filter the involuntary movements of the subjects.

Prediction by Neural Networks

An application was developed with the goal of predicting the advancement of diabetes in an individual by analyzing their corresponding heatmap (Additional materials 7). The input was given as an image consisting of 10 rows and 20 columns of colored rectangles. I our case the image ratio was not important, as the application selected only the color at the center of each rectangle (width/40, height/20) (in increments of width/20 and height/10 respectively). The selected color matrix was then transformed into a matrix of depth 1 which contained values between 0 and 1. This transformation was done by reversing the 3-color-scale conversion previously used to create the picture. The application then transmitted the data to a python script, along with the model, and the script returned a value which has been converted to a percentage, where 0% meant healthy and 100% meant diabetes.

The python script loads a neural network and returns the output of this network when it receives the input data. The application can also be used to train a new neural network model. The C# code reads images from a selected folder and converts them to the desired depth-1 matrices, then runs the python script which trains the new model. The model can then be saved under a different name and loaded whenever necessary.


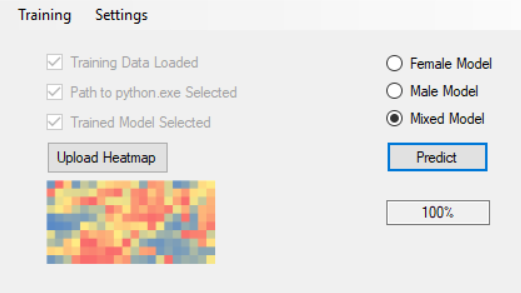


There were 36 images used for the training of the current model (Additional materials 8). These are divided into: 5 healthy female patients, 8 diabetic female patients, 13 healthy male patients and 10 diabetic male patients. Multiple models achieved 100% accuracy on the training data. Each model is trained in 3 ways: once only on female patients, once only on male patients and finally on all the data. The male and female models are more accurate when the gender of the person whose heatmap is being input is known.

The configuration of the current model is presented below:

1. A 2D Convolution [3x3] layer
2. A Dropout layer (to avoid overfitting)
3. A MaxPooling [2x2] layer

This structure is repeated 3 times, each time increasing the number of neurons in order to detect larger features. The MaxPooling layer is not needed or used in the 3^rd^ repetition.

1. A Flattening Layer
2. A RELU dense layer (256 neurons)
3. A RELU dense layer (128 neurons)
4. A Sigmoid output layer

At the moment there is no training data in-between 0% and 100%, therefore the intermediate values can only be considered as trust value of the network, under 50% meaning healthy and over 50% meaning different degrees of predisposition to diabetes. The network can, however, be trained on intermediate data in the future. This will allow it to predict with more accuracy the stage of diabetes of an individual.

The following picture is the representation of the network as output by the Keras Deep Learning Library:


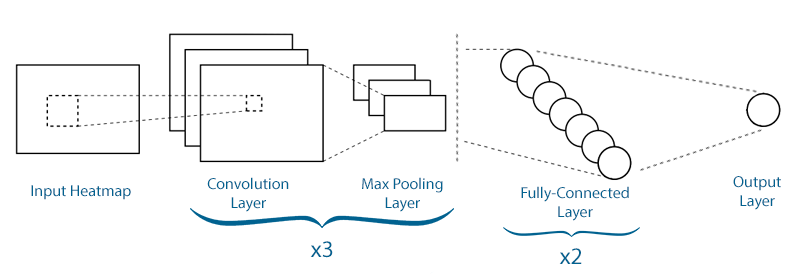


The first column represents the layer type, the second is the output shape of the layer and the third is the number of weights created by the layer:


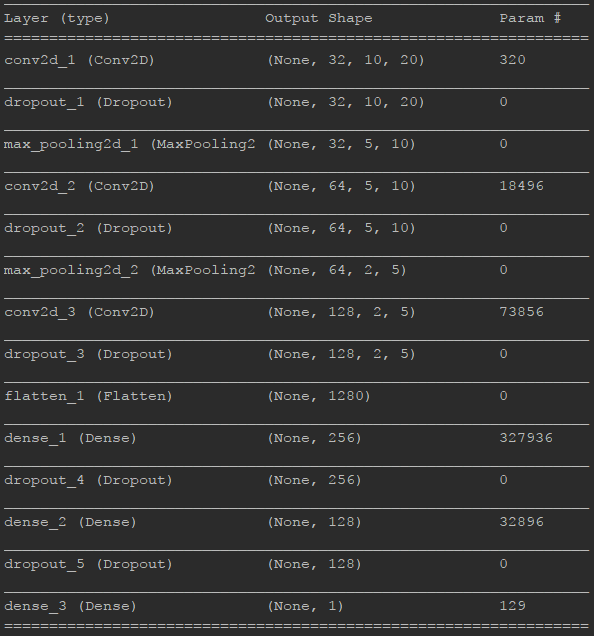


1. The conv2d layer is a Convolution Layer. The primary purpose of this layer is to extract features from the input image. The convolution layer takes a 3x3 matrix called a filter and slides it over the input matrix. At each step, the values of the filter are multiplied with the corresponding values in the input matrix and added together, then inserted in a new matrix. The shape of the new matrix remains the same as that of the initial matrix. In the case of the first layer, (None, 32, 10, 20) it means that there are 32 neurons, each having an input of 10 rows and 20 columns.
2. The goal of the dropout layer is to prevent overfitting. By choosing a random set of neurons that will not activate at each training step, the dropout layer prevents the network from learning the training set exclusively.
3. The max_pooling2d layer is a Max Pooling Layer. This layer slides a 2x2 square over the input matrix with a step of 2 and selects the maximum of the 4 values, inserting it into the output matrix. This effectively halves the width and the height of the input matrix, in this case the shape becoming 5x10.
4. By repeating this structure, the convolution layers are able to detect larger features on the image. The Max Pooling Layer is not repeated a 3^rd^ time.
5. The flatten layer deconstructs the shape of the input matrix and outputs all the values of the input in a single array. In this case, the input layer had 128 neurons, each outputting a matrix of 2x5. Therefore, the flattened layer has 128*2*5=1280 values.
6. Dense layers are the basic neural network building block, taking all the inputs and fully connecting them to all the outputs with various weights.

Instructions for the prediction process (Windows):

1. Unpack the archive.
2. Install python 3.6.x
3. Install the following python packages:

- numpy
- tensorflow
- keras
- skLearn
- h5py

1. Run the executable file HeatmapAnalysis.exe
2. In [Settings -> Select python.exe Path], navigate to the python instalation and select the python.exe file.
3. In [Training -> Load Model], select any of the json files starting with Final (Final_A, Final_F or Final_M).
4. Click on [Upload Heatmap] and select an image file in a supported format.
5. Select a model from the 3 options on the right side of the screen, then click [Predict].

To train a model on your own data, go through the first 5 steps described above, then:

1. Create a folder in which all the heatmap images will be stored. The file name is formed as follows: The first letter represents wether the patient is female (F) or male (M). The second symbol is either minus (-) or plus (+), representing the diagnostic of the patient (minus for healthy, plus for diabetic). The rest of the name is the index of the image, with no leading zeros. The extension is .bmp. (”F-0.bmp”, ”F-1.bmp”, ”F+0.bmp”, ”M-0.bmp”, ”M+0.bmp").
2. In [Training -> Load Training Data] select the folder containing the heatmaps.
3. Select [Training -> Train Model]. The training will take several minutes.
4. Finally, use [Training -> Save Model] to save the trained model under a different name.
